# Combination of mean CT value and maximum CT value as a novel predictor of lepidic predominant lesions in small lung adenocarcinoma presenting as solid nodules

**DOI:** 10.1038/s41598-022-09173-1

**Published:** 2022-03-31

**Authors:** Satoshi Koezuka, Atsushi Sano, Yoko Azuma, Takashi Sakai, Keiko Matsumoto, Nobuyuki Shiraga, Tetuo Mikami, Naobumi Tochigi, Yoshitaka Murakami, Akira Iyoda

**Affiliations:** 1 Division of Chest Surgery, Department of Surgery, Toho University School of Medicine, 6-11-1 Omori-nishi, Ota-ku, Tokyo, 143-8541 Japan; 2 Department of Radiology, Toho University School of Medicine, 6-11-1 Omori-nishi, Ota-ku, Tokyo, 143-8541 Japan; 3 Department of Pathology, Toho University School of Medicine, 5-21-16 Omori-nishi, Ota-ku, Tokyo, 143-8540 Japan; 4 Department of Surgical Pathology, Toho University School of Medicine, 6-11-1 Omori-nishi, Ota-ku, Tokyo, 143-8541 Japan; 5 Departmant of Medical Statistics, Toho University, 5-21-16 Omori-nishi. Ota-ku, Tokyo, 143-8540 Japan

**Keywords:** Cancer, Oncology

## Abstract

Lung adenocarcinomas presenting as solid nodules are occasionally diagnosed as lepidic predominant lesions. The aim of this study was to clarify the histological structure and to identify factors predictive of lepidic predominant lesions. We retrospectively reviewed 38 patients that underwent lobectomy for small (≤ 2 cm) adenocarcinoma presenting as solid nodules. Resected tumor slides were reviewed and histological components were evaluated. Clinical and radiological data were analyzed to identify factors predictive of lepidic predominant lesions. Of 38 solid nodules, 9 (23.7%) nodules were lepidic predominant lesions. Five-year disease-free survival (DFS) rates were 100% for lepidic predominant lesions (n = 9) and 74.6% for non-lepidic predominant lesions (n = 29). Mean CT values (*p* = 0.039) and maximum CT values (*p* = 0.015) were significantly lower in lepidic predominant lesions compared with non-lepidic predominant lesions. For the prediction of lepidic predominant lesions, the sensitivity and specificity of mean CT value (cutoff, − 150 HU) were 77.8% and 82.8%, respectively, and those of maximum CT value (cutoff, 320 HU) were 77.8% and 72.4%, respectively. A combination of mean and maximum CT values (cutoffs of − 150 HU and 380 HU for mean CT value and maximum CT value, respectively) more accurately predicted lepidic predominant lesions, with a sensitivity and specificity of 77.8% and 86.2%, respectively. The prognosis of lepidic predominant lesions was excellent, even for solid nodules. The combined use of mean and maximum CT values was useful for predicting lepidic predominant lesions, and may help predict prognosis.

## Introduction

Lung cancer is the third most common cancer in Japan and the leading cause of cancer-related mortality, despite the development of new therapies^1^. Adenocarcinoma is the most common histological subtype of lung cancer. Pathologically, adenocarcinomas are classified as preinvasive lesions, minimally invasive adenocarcinoma (MIA), invasive adenocarcinoma, or variants of invasive adenocarcinoma. Invasive adenocarcinoma is further classified into specific subtypes according to the predominant pattern. Lepidic predominant lesions, such as adenocarcinoma in situ (AIS), MIA, and lepidic predominant invasive adenocarcinoma, are associated with excellent survival^2^.

In recent years, limited resection for peripheral early-stage lung cancer is sometimes performed^3,4^. Radiologically, adenocarcinoma is composed of various proportions of ground-glass opacity (GGO) and solid opacity. Nodules are classified as either pure ground-glass nodules, part-solid nodules, or solid nodules depending on the presence or absence of GGO and solid components^5^. In general, GGO findings on computed tomography (CT) tend to reflect a histological lepidic component, while solid opacity on CT tends to reflect invasive histological components^2^. CT findings indicating solid nodules have generally been predictive of a diagnosis of invasive adenocarcinoma and a poorer prognosis compared with lesions with GGO components^6,7^. However, contrary to predictions based on CT findings, solid nodules are sometimes revealed to be lepidic predominant lesions on histological analysis. The histological features of solid nodules remain unclear, and preoperative prediction of solid nodule histological subtype is challenging.

Elucidation of the histological structure of adenocarcinoma presenting as solid nodules may allow more precise prediction of prognosis and may be helpful for the determination of appropriate surgical procedures. The objectives of the present study are to clarify the histologic features of solid nodules and to find a way of predicting solid nodules correlate with a favorable prognosis.

## Patients and methods

The present study was approved by the Ethics Committee of Toho University School of Medicine (No. A21018_A18021_27085). The research plan was published on the hospital homepage, along with an opt-out option. We disclosed the content of this study and provided an opportunity for patients to refuse to participate in this study. Since this study was a retrospective study, a waiver of informed consent was granted by Ethics Committee of Toho University School of Medicine. All methods were carried out in accordance with the Declaration of Helsinki.

Between 2008 and 2016, 38 lesions in 38 patients who underwent lobectomy for small (≤ 2 cm) lung adenocarcinomas presenting as solid nodules on CT were evaluated retrospectively. Medical records were reviewed to evaluate clinical characteristics, radiological findings, histological findings, and prognoses.

**Figure 1:**
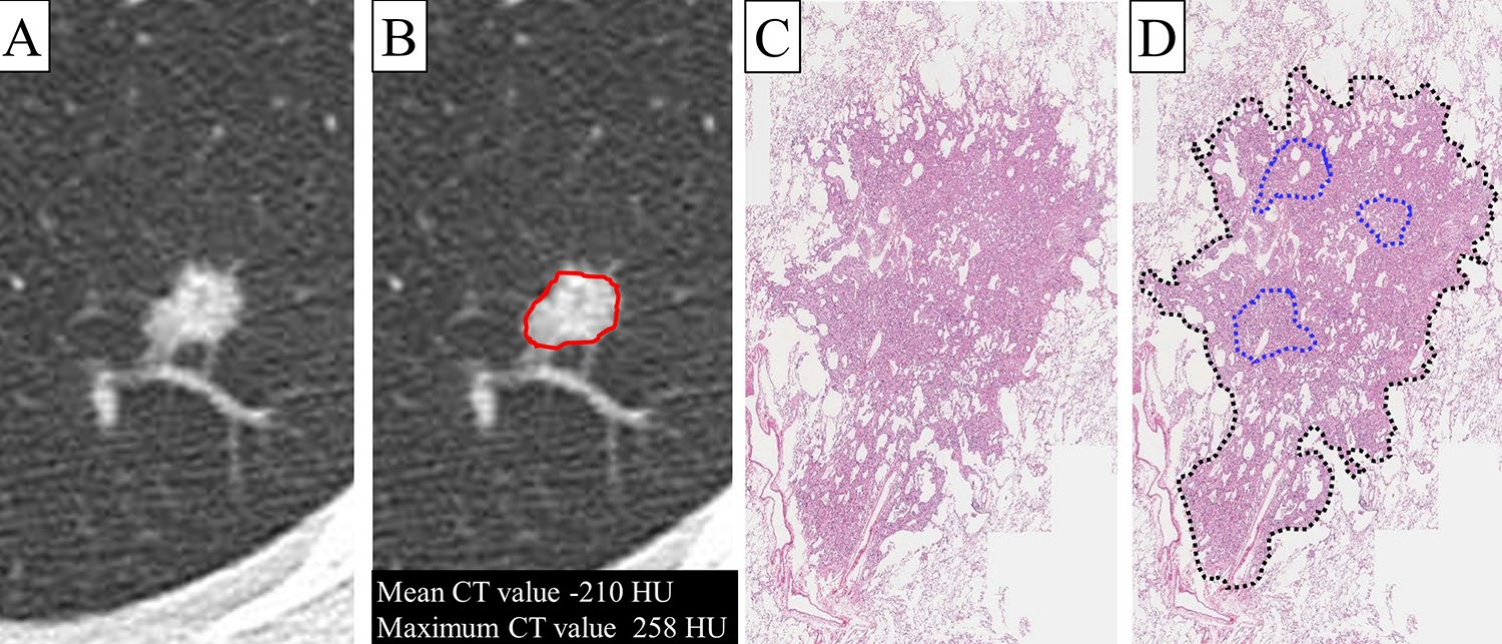
Representative case of a small lung adenocarcinoma presenting as solid nodule. (**A**) Axial CT image showing a solid nodule. (**B**) The region of interest (ROI) was manually placed using AZE Virtual Place software. (**C**) This lesion revealed lepidic predominant histology. (**D**) Three acinar components were observed in the central area (marked by blue dots), and a lepidic component was seen surrounding the acinar component.

CT studies performed at our institution were reviewed. The CT scans were performed on a 64-detector CT row scanner (Aquilion, Toshiba, Japan or Somatom Definition Flash, Siemens, Germany). Parameters for the 64-detector CT row scanner (Aquilion, Toshiba, Japan) were as follows: 1.0 mm slice thickness, 2.0 mm interval, 1.0 mm collimation, 0.5-s gantry rotation time, 120 kVp, FC52 standard reconstruction kernel. Parameters for the 64-detector CT row scanner (Somatom Definition Flash, Siemens, Germany) were as follows: 1.0 mm slice thickness, 2.0 mm interval, 1.0 mm collimation, 0.5-s gantry rotation time, 120 kVp, B70 standard reconstruction kernel. All CT images were displayed with lung window settings (window width, 1600 Hounsfield units [HU]; window level, − 600 HU). These CT protocols are identical to those used in our previous study^8^. All CT images were reviewed by a single radiologist with 17 years of experience. The mean and maximum CT values of the solid region were calculated using AZE Virtual Place (AZE Ltd, Tokyo, Japan). For determination of the mean and maximum CT values, a region of interest (ROI) was manually placed over the entire tumor in the slice of the maximum area (Fig. 1A,B). The correlation between CT values and histologic predominant subtypes was evaluated. Prediction probability for lepidic predominant lesions was analyzed using a receiver operating characteristic (ROC) curve.

Histological analysis was performed in the same way as in our previous study^8^. Histological components (acinar, lepidic, micropapillary, papillary, solid, pattern of variants, bronchi, cavities, edema, fibrosis, inflammation, necrosis, pleural thickening, and vessels) within tumors were evaluated. All histological findings were reviewed by two observers, one of whom is a pathologist (T.M), and other is a surgeon (S.K). In addition, the area of each histological component was analyzed using digital image files and ImageJ software (National Institutes of Health, Bethesda, MD, USA) (Fig. 1C,D). The occupancy ratio of each component within the same tumor was recorded in 0.1% increments. Histological components other than acinar, lepidic, micropapillary, papillary, solid, pattern of variants, fibrosis, and necrosis were classified as ‘other components’. All lesions were classified into predominant histological subtypes according to the predominant pattern. When lepidic component occupied the highest proportion of the entire tumor, the tumor was defined as lepidic predominant adenocarcinoma. In this study, the lepidic predominant subtype included AIS, MIA, and lepidic predominant invasive adenocarcinoma. All lesions were divided into two subgroups: lepidic predominant and non-lepidic, which included acinar, micropapillary, papillary, solid, and variants.

### Statistical analysis

The clinical, radiological, and histological difference between lepidic and non-lepidic predominant lesions were compared by using chi-squared test or Fisher’s exact test in dichotomous variables and Student’s *t* test or Mann–Whitney *U* test in continuous variables. Diagnostic ability in mean and maximum CT values for detecting lepidic/non-lepidic predominant lesions were examined using sensitivity and specificity. Area under the receiver operating characteristics (ROC) curve was calculated by evaluating the diagnostic ability of mean and maximum CT values. Disease-free survival (DFS) was defined as the time between the date of surgery and the date of recurrence or death. DFS was analyzed using the Kaplan–Meier method and compared by log-rank test. *p* values < 0.05 were considered to indicate statistical significance. All statistical analyses were performed using SPSS software version 22.0 (SPSS Inc; Chicago, IL, USA).

## Results

Clinical, radiological, and histological characteristics are summarized in Table 1. Of all lesions, 9 (23.7%) were lepidic predominant lesions; all 9 were non-mucinous. The proportion of lepidic components in lepidic predominant adenocarcinoma ranged from 31.9 to 88.6%. Histological findings of each histological component are summarized in Table 2. Lepidic components were present in 18 (47.4%) nodules. The areas of lepidic components were the second largest among the histological components, and the occupancy ratios of lepidic components were the third highest among the histological components.

**Table 1:** Clinical, radiological, and histological characteristics.

Characteristics	n (%) or mean ± SD (range)	
Age (years)	63.7 ± 10.9	(38–84)
**Sex**		
Men	29	(76.3)
Women	9	(23.7)
**Smoking status**		
Never	6	(15.8)
Former/current	32	(84.2)
CEA (ng/ml)	3.0 ± 8.2	(0.7–47.6)
SLX (U/ml)	32.3 ± 7.2	(20.4–57.0)
Tumor size on CT (mm)	15 ± 3	(8–20)
Maximum CT value (HU)	345 ± 89	(200–625)
Mean CT value (HU)	− 88 ± 109	(− 383 to 85)
**Clinical stage (8th edition)**		
IA1	3	(7.9)
IA2	35	(92.1)
**Predominant histological subtypes**		
Lepidic	9	(23.7)
Papillary	15	(39.5)
Acinar	4	(10.5)
Solid	7	(18.4)
Variants (colloid)	3	(7.9)
**Pathological nodal involvement**		
Negative	37	(97.4)
Positive	1	(2.6)
**Pathological stage (8th edition)**		
0	1	(2.6)
IA1	17	(44.7)
IA2	12	(31.6)
IA3	1	(2.6)
IB	6	(15.8)
IIB	1	(2.6)

Categorical variables are shown as numbers (%). Continuous variables are presented as mean ± SD (range). *CEA* carcinoembryonic antigen, *SLX* sialyl Lewis X.

**Table 2:** Histological features of each histological component.

Histological component	n (%)	Occupy ratio*, %		Area, mm^2^	
Lepidic	18 (47.4)	16.5 ± 26.0	(0–88.6)	12.1 ± 20.7	(0–88.8)
Papillary	26 (68.4)	37.0 ± 36.9	(0–100.0)	33.1 ± 43.2	(0–152.6)
Acinar	19 (50.0)	12.3 ± 22.7	(0–100.0)	8.9 ± 16.7	(0–78.0)
Solid	12 (31.6)	17.4 ± 34.2	(0–100.0)	10.6 ± 25.4	(0–125.6)
Micropapillary	3 (7.9)	0.5 ± 2.0	(0–11.5)	0.4 ± 1.9	(0–11.9)
Colloid	3 (7.9)	6.4 ± 22.7	(0–100.0)	3.9 ± 16.9	(0–84.0)
Fibrosis	13 (34.2)	7.4 ± 13.8	(0–48.6)	4.7 ± 8.0	(0–29.4)
Necrosis	1 (2.6)	0.3 ± 1.9	(0–11.5)	0.3 ± 1.9	(0–12.0)
Others	5 (13.2)	2.1 ± 8.3	(0–45.9)	2.1 ± 8.3	(0–45.9)

*The ratio of histological component within the tumor for each of the 38 lesions.

Continuous variables are presented as mean ± SD (range).

**Figure 2:**
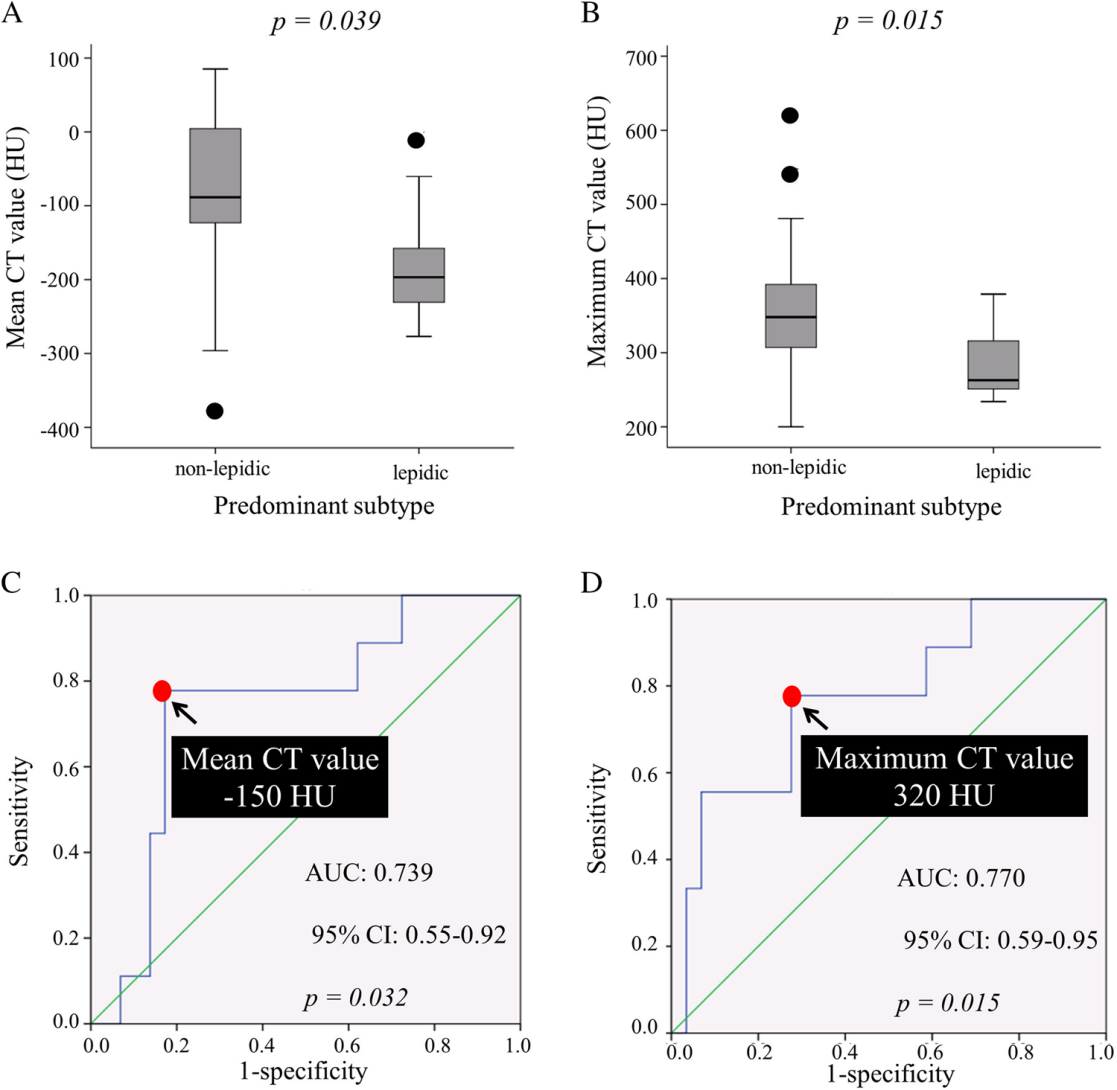
(**A**,**B**) Comparison of CT values between lepidic and non-lepidic predominant lesions. (**A**) Lepidic predominant lesions showed significantly lower mean CT values. (**B**) Lepidic predominant lesions showed significantly lower maximum CT values. (**C**,**D**) Receiver operating characteristic curve analysis for lepidic predominant lesion prediction. (**C**) The sensitivity and specificity of mean CT values were 77.8% and 82.8%, respectively. (**D**) The sensitivity and specificity of maximum CT values were 77.8% and 72.4%, respectively.

Figure 2A compares the mean CT values of lepidic and non-lepidic predominant lesions. Mean CT values were significantly lower in lepidic predominant lesions (median, − 196 ± 85 HU) compared with non-lepidic predominant lesions (median, − 88 ± 109 HU; *p* = 0.039). Figure 2B shows maximum CT values of lepidic and non-lepidic predominant lesions. The maximum CT value was significantly lower in lepidic predominant lesions (median, 263 ± 53 HU) than in non-lepidic predominant lesions (median, 348 ± 91 HU; *p* = 0.015). As shown in Fig. 2C, the optimal threshold mean CT value for lepidic predominant lesions was − 150 HU (area under the ROC curve, 0.739; 95% CI, 0.55–0.92), with a sensitivity of 77.8% and a specificity of 82.8%. Figure 2D shows that the optimal threshold maximum CT value for lepidic predominant lesions was 320 HU (area under the ROC curve, 0.770; 95% CI, 0.59–0.95), with a sensitivity of 77.8% and a specificity of 72.4%.

**Figure 3:**
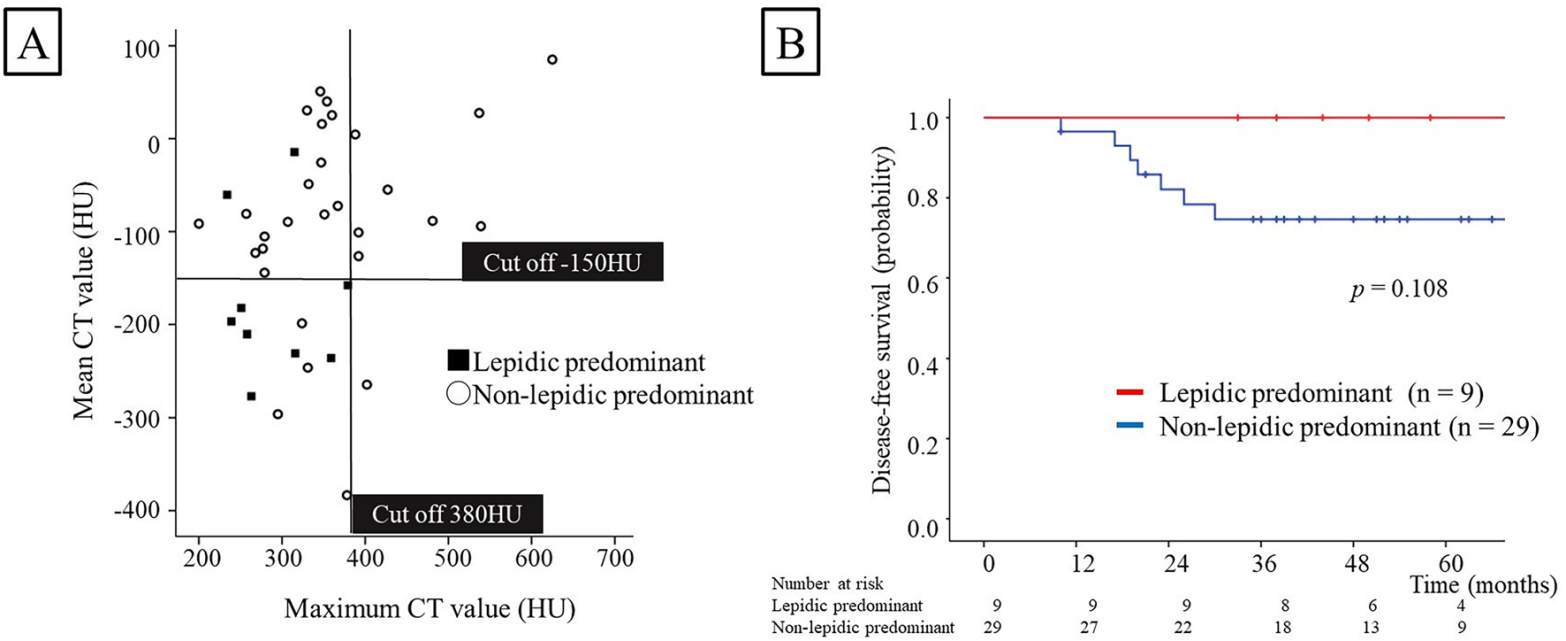
(**A**) Relationship between the mean and maximum CT values. The lines indicate the optimal cutoff value for the mean CT value (− 150 HU) and the maximum CT value (380 HU). The sensitivity and specificity for lepidic predominant lesions were 77.8% and 86.2%, respectively. (**B**) Kaplan–Meier survival curves for disease-free survival (DFS). Patients with lepidic predominant lesions experienced no recurrences.

Figure 3A shows the relationship between the mean and maximum CT values. To predict lepidic predominant lesions more accurately, we analyzed the optimal thresholds for the mean and maximum CT values based on the marks shown in Fig. 3A. The optimal threshold values for lepidic predominant lesions were − 150 HU for mean CT value and 380 HU for maximum CT value (95% CI, 0.64–0.99), with a sensitivity of 77.8% and a specificity of 86.2%.

Table 3 presents differences in clinical and radiological characteristics between lepidic predominant and non-lepidic predominant lesions. No significant differences were observed between lepidic and non-lepidic predominant lesions.

**Table 3:** Comparison of clinical and radiological characteristics between lepidic predominant and non-lepidic predominant lesions.

Characteristics	Predominant Subtypes		*p* value
	Lepidic	Non-lepidic	
**Sex**			
Men	6 (66.7)	23 (79.3)	0.655
Women	3 (33.3)	6 (20.7)	
Age (years)	61.2 ± 10.7	64.5 ± 11.0	0.429
**Smoking status**			
Never	3 (33.3)	3 (10.3)	0.131
Former/current	6 (66.7)	26 (89.7)	
CEA (ng/ml)	2.4 ± 1.1	6.4 ± 9.1	0.142
SLX (U/ml)	32.1 ± 11.0	32.4 ± 5.7	0.325
Tumor size on CT (mm)	13.7 ± 2.9	15.6 ± 3.0	0.106

Categorical variables are shown as numbers (%). Continuous variables are presented as mean ± SD (range). *CEA* carcinoembryonic antigen, *SLX* sialyl Lewis X. *p* value in Chi-square test or Fisher’s exact test, Student’s *t* test, and Mann–Whitney *U* test.

The median follow-up time of 38 patients was 51.5 months (range, 10–118 months). The 5-year DFS rate was 80.9%. Recurrences were observed in 6 patients, all of whom had papillary predominant lesions. No recurrences were observed among patients with lepidic predominant lesions. The DFS analysis did not reveal significant differences between lepidic predominant and non-lepidic predominant lesions [5-year DFS rate, 100.0% vs 74.6%; *p* = 0.108 (Fig. 3B)].

## Discussion

The present study aimed to identify the clinical factors correlated with a favorable prognosis in small (≤ 2 cm) lung adenocarcinomas presenting as solid nodules. According to the current (8th) edition of the TNM Classification, solid components observed on CT are defined as invasive components^9^. Interestingly, we observed that among all histological components, the areas of lepidic components were the second largest and the occupancy ratios were the third highest. Furthermore, we showed that mean CT values and maximum CT values were significantly lower in lepidic predominant lesions compared with non-lepidic predominant lesions. Moreover, combined use of mean and maximum CT values was useful in predicting the lepidic predominant lesions associated with an excellent prognosis, even for solid nodules.

The first point I would like to emphasize in this study is its detailed analysis of the histological components of solid lung nodules. Only one study assessed the internal histological components of solid nodules, but no detailed results were reported^7^. The second point I would like to emphasize in this study is the use of CT values to evaluate histological subtypes. To the best of our knowledge following research of the PubMed database, this is the first study to evaluate the correlation between histological subtypes and CT values, with a focus on solid nodules.

Solid nodules are considered to be highly invasive lesions. Even among small-sized solid nodules, postoperative nodal involvement is observed in approximately 4–26% of cases^4,10,11^. A high incidence (20%) of locoregional recurrence following limited resection of small-sized solid nodules has been observed^12^. Preoperative prediction of postoperative prognosis is important for selection of surgical procedures. As in previous reports^10^, we showed that lepidic predominant lesions are associated with a good prognosis, even for solid lesions. Therefore, identification of lepidic predominant lesions is essential for predicting prognosis in this patient population.

Previous studies reported that lepidic predominant lesions were correlated with low serum CEA levels^13^, low SUVmax values^10,14,15^, low 75th percentile CT attenuation values, and maximum CT values^16^. Only one study has previously evaluated the clinical features of lepidic predominant adenocarcinomas presenting as solid nodules^10^. These investigators reported that a lower SUVmax value was a significant clinical feature. However, Iwano et al. reported that solid-type primary lung cancers with lesions ≤ 2 cm tend to have false-negative PET findings^17^. Therefore, we should consider the possibility of false-negative PET findings when evaluating small-sized lesions.

In the present study, we demonstrated that the combination of mean and maximum CT values is useful for predicting lepidic predominant lesions in small lung adenocarcinoma presenting as solid nodules. CT values reflect both intratumoral cellularity and density. In our previous study, we compared radiological and histological findings of pure GGO lesions with heterogeneous density, the invasive component was present in the site with high CT value and the lepidic component was present in the site with low CT value^8^. Further, adenocarcinomas presenting as pure GGO lesions containing invasive components and STAS-positive adenocarcinomas demonstrated a strongly higher CT value. Therefore, the maximum CT value is considered to be a reliable factor for predicting invasiveness. Some prior reports demonstrated that lepidic components with collapsed alveolar spaces and mucus sometimes appear as solid components on CT^18,19^. Therefore, the CT value of lepidic predominant lesions presenting as solid nodules is considered to be lower than that for non-lepidic predominant lesions. CT values inside adenocarcinoma are diverse, reflecting heterogeneous histological components^16^. We think the mean CT value reflects the histological findings of the whole tumor. To improve accuracy, we assessed the combination of mean and maximum CT values. In an analysis based on the respective cutoffs of the mean CT value of − 150 HU and the maximum CT value of 320 HU, we were not able to accurately divide lepidic predominant lesions. Therefore, we used the combination of the mean CT value of − 150 HU and the maximum CT value of 380 HU, and were able to differentiate lepidic predominant lesions from non-lepidic predominant lesions more accurately than if these CT values were used individually. Because both the maximum and mean CT values are easily measured and can be used in clinical practice, they were selected as ideal indicators.

The present study had some limitations. First, this study included a small number of cases. It is necessary to analyze more cases and verify the results of this study in the future. Second, the largest slice on CT sometimes cannot reflect the properties of the entire tumor due to the intratumoral heterogeneity. We think CT volume measurement reflects the nature of the tumor more than the single-slice CT evaluation. On the other hand, the analysis of CT volume measurement is so complicated with a currently-used DICOM viewer that it is not easy to use in clinical practice. Therefore, we tried this analysis with single-slice CT images. In addition, further studies are needed to identify an optimal cutoff value that is more sensitive for distinguishing lepidic predominant lesions. Nevertheless, we think that the present results will be useful in predicting postoperative prognosis.

## Conclusions

This study demonstrated that lepidic components accounted for a relatively large proportion of small lung adenocarcinomas presenting as solid nodules. The prognosis of lepidic predominant lesions tends to be better than that of non-lepidic lesions, even for solid nodules. The combined use of mean and maximum CT values can be useful for predicting lepidic predominant lesions in solid nodules. Based on the results of this study, combined use of mean and maximum CT values may help predict the prognosis of solid nodules and contribute to a choice of surgical procedures.
